# All-Solution-Processed Quantum Dot Light-Emitting Diode Using Phosphomolybdic Acid as Hole Injection Layer

**DOI:** 10.3390/ma16041371

**Published:** 2023-02-06

**Authors:** Jeong Ha Hwang, Eunyong Seo, Sangwook Park, Kyungjae Lee, Dong Hyun Kim, Seok Hyoung Lee, Yong Woo Kwon, Jeongkyun Roh, Jaehoon Lim, Donggu Lee

**Affiliations:** 1 Department of Semiconductor Engineering, Gyeongsang National University, Jinju 52828, Republic of Korea; 2 Department of Electrical Engineering, Pusan National University, Busan 46241, Republic of Korea; 3 Department of Energy Science, Center for Artificial Atoms, Sungkyunkwan University (SKKU), Suwon 16419, Republic of Korea

**Keywords:** phosphomolybdic acid, solution processing, hole injection layer, quantum dot, light-emitting diode

## Abstract

In this study, we investigate phosphomolybdic acid (PMA), which allows solution processing of quantum dot light-emitting diodes. With its low cost, easy solution processes, and excellent physical and optical properties, PMA is a potential candidate as the hole injection layer (HIL) in optoelectronic devices. We evaluate the physical and electrical properties of PMA using various solvents. The surface morphology of the PMA film was improved using a solvent with appropriate boiling points, surface tension, and viscosity to form a smooth, pinhole-free film. The energy level was regulated according to the solvent, and PMA with the appropriate electronic structure provided balanced charge carrier transport in quantum dot electroluminescent (QD-EL) devices with enhanced efficiency. A device using PMA dissolved in cyclohexanone was demonstrated to exhibit improved efficiency compared to a device using PEDOT:PSS, which is a conventional solution HIL. However, the stability of PMA was slightly poorer than PEDOT:PSS; there needs to be further investigation.

## 1. Introduction

Colloidal quantum dots (QDs), which have tunability, high color purity, and high photoluminescence quantum yield, have attracted wide attention and recently realized commercial applications in display technology [1,2,3,4,5,6,7]. The commercial applications are based on photoluminescence (PL), involving the color conversion of blue backlight to red and green [8,9,10]. However, QD-electroluminescent (EL) devices are still under development. Recently, tremendous efforts in various research fields have resulted in highly efficient EL devices with over 20% external quantum efficiency (EQE), which is comparable with those of organic light-emitting diodes (LEDs) [11,12,13,14,15].

Typically, QD-EL devices have a hybrid structure comprising an organic hole injection/transport layer and an inorganic electron injection/transport layer. Poly(3,4-ethylenedioxyth-iophene):poly(styrene-sulfonate) (PEDOT:PSS) is widely used as a hole injection layer (HIL) in QD-EL devices because of its moderate work function, high conductivity, transparency, and solution processability. However, PEDOT:PSS is known for stability issues due to its acidic and hygroscopic properties [16].

As an alternative to solution-processed HILs, transition-metal oxides have been investigated using various thin-film fabrication methods, such as dispersion of nanoparticles [17,18,19], the sol–gel method [20,21,22,23,24,25,26], and dissolution of metal oxide powder [27]. Moreover, MoO_3_ [17,18,20,21], CuO [22], NiO_x_ [23,24], VO_x_ [25,26], and WO_3_ [27] are generally investigated as HIL for organic electronics and QD-EL devices. Nevertheless, more research is still needed to develop the solution-based HIL to attain high stability and efficiency.

Recently, phosphomolybdic acid (PMA) [28,29,30,31,32,33], which is a heteropoly acid containing MoO_3_, has been reported to work as HIL in QD and organic electronics. Since PMA is soluble in polar solvents, it has process orthogonality with organic materials, which have typically nonpolar characteristics. Q. Wu et al. investigated the polarity effect of the solvent of PMA solution on the performance of a QD-EL device on top of a polymeric hole transport layer (HTL) [28]. Zhu et al. applied the PMA as a hole-extraction layer of organic photovoltaics, which exhibited a similar performance to MoO_3_ [29]. In addition, Pu et al. reported a charge-generation layer of solution-based tandem OLED with a structure of ZnO/polyethyleneimine-ethoxylated/PMA [30].

Another issue for solution-processed thin-film devices is obtaining high-quality films with a uniform, low-surface-roughness and pinhole-free morphology. The quality of solution-processed thin film differs significantly from the processing solvent due to its dry condition changes with solvent properties such as dry speed and surface tension. Moreover, PMA is known for varying the electronic property depending on the process condition. S. Ohisa et al. reported that PMA thin-film processing conditions such as annealing temperature and processing atmosphere affect the reduction of Mo, which can act as an oxygen vacancy; consequently, the work function is also changed depending on the processing conditions [31].

In this study, we investigated the optimal solvent for PMA as the HIL of a standard-structured QD-EL device under nitrogen atmosphere. We selected four solvents for the PMA process: acetonitrile, n-butanol, cyclohexanone, and dimethyl sulfoxide (DMSO). We found that the process solvent strongly affects work function, surface morphology, and oxygen vacancy. QD-EL devices were fabricated with different solvents of PMA, and cyclohexanone exhibited optimal device performance.

## 2. Materials and Methods

### 2.1. Materials

Chlorobenzene (CB, anhydrous, 99.8%), cyclohexanone (ACS reagent, ≥99.0%), 1,4-dioxane (anhydrous, 99.8%), dimethyl sulfoxide (DMSO, ≥99.9%), acetonitrile (anhydrous, 99.8%), n-butanol (anhydrous, 99.8%), phosphomolybdic acid (PMA), and poly(9-vinylcarbazole) (PVK) were purchased from Sigma Aldrich. Furthermore, N4,N4′-di(Naphthalen-1-yl)-N4,N4′-bis(4-vinylphenyl)biphenyl-4,4′-diamine (VNPB) was purchased from OSM. In addition, we prepared QD solutions (CdSe/CdS/CdZnS) according to a previous report [34].

### 2.2. QD-EL Device Fabrication

QD-EL devices were fabricated on glass substrates with patterned indium tin oxide (ITO). The substrates were then washed with deionized water (DI), acetone, and isopropyl alcohol (IPA) for 10 min each and then dried in a vacuum oven for at least 30 min. The ITO substrates were then ultraviolet (UV)–ozone treated for 15 min, and 3.0 mg/mL of PMA was spin-coated at 6000 rpm for 45 s on the ITO glass. Thereafter, the coated substrates were baked at 180 °C for 20 min on a hot plate. The thickness of the PMA films was estimated to be below 10 nm. After annealing, ~10 mg/mL of VNPB solution in CB was spin-coated at 3000 rpm for 45 s and annealed at 200 °C for 1 h. Next, ~1.0 mg/mL of PVK solution in CB was spin-coated and baked at 150 °C for 15 min. Then, ~10 mg/mL of the CdSe/CdZnSe/ZnSeS QD solution in octane was spin-coated and baked at 120 °C for 30 min. Subsequently, ~20 mg/mL of ZnMgO solution in n-Butanol was spin-coated and baked at 120 °C for 30 min. All spin coatings were performed in N_2_ atmosphere. Finally, all the substrates were transferred to a vacuum chamber, and Ag was thermally evaporated at a rate of 1.0 Å/s at ~10^−7^ Torr.

### 2.3. Hole-Only Device Fabrication

The hole-only device was fabricated on an ITO substrate in the same method as the QD-EL device fabrication process. The PMA, VNPB, and PVK were spin-coated in the same condition as the QD-EL device fabrication method. Then, all the substrates were transferred to a vacuum chamber, and MoO_3_ was thermally evaporated at a rate of 0.5 Å/s. Finally, Ag was thermally evaporated at a rate of 1.0 Å/s. All vacuum thermal evaporation was performed in an atmosphere of ~10^−7^ Torr.

### 2.4. Thin Film and Device Characterization

The surface topographies of PMA with different solvents were obtained using an atomic force microscope (NX10, Park-Systems, Suwon, Korea). The thicknesses of the thin films were measured with ellipsometry (SE MG-1000, Nanoview, Ansan, Korea). The UV–visible absorption spectra were measured with a UV–VIS spectrometer (UV-2600i, Shimadzu, Kyoto, Japan). The work function analysis was performed with an X-ray Photoelectron Spectrometer (AXIS SUPRA, Shimadzu) using a He-I discharge lamp. Contact angle measurement was performed using the SEO Phoenix 300 contact angle analyzer. Furthermore, the QD-EL device performances were characterized using a Keithley (2400, Keithley, Cleveland, OH, USA) source measurement unit and a spectroradiometer (PR-655, JADAK, North Syracuse, NY, USA).

## 3. Results and Discussion

### 3.1. Thin-Film Characteristics of PMA

**Figure 1:**
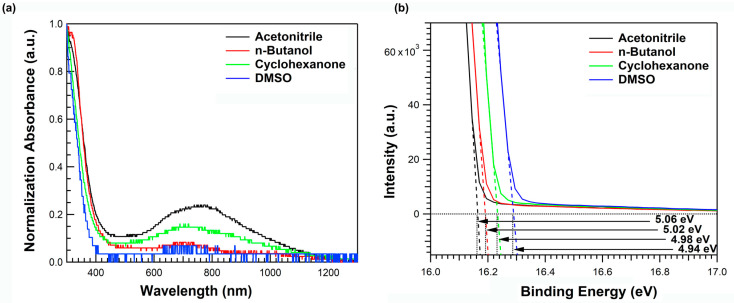
Thin-film characteristics of PMA with various solvents: (**a**) UV–Vis–NIR absorbance spectrum, (**b**) Cut-off region fitting graph of UPS measurement.

We fabricated a PMA thin film by spin-casting with different solvents, namely, acetonitrile, n-butanol, cyclohexanone, and DMSO, in a nitrogen glovebox with an annealing temperature of 180 °C. Figure 1a shows the normalized UV–visible–near-infrared (UV–Vis–NIR) absorption spectra of PMA thin films with different solvents; the main absorption peak occurs at a wavelength below 400 nm, and the broad absorption in the NIR region is attributed to the oxygen vacancy, which originates from the reduction of Mo(VI) [31]. Because the PMA film fabrication process was executed under an inert atmosphere, acetonitrile, which has no oxygen atom, exhibited the strongest absorption intensity in the NIR region.

In addition, these chemical composition changes affected the electrical properties of PMA films. Therefore, we evaluated the work function of the PMA films using UV photoelectron spectroscopy (UPS) (Figure 1b). A He-I (hv = 21.22 eV) excitation line was used for UPS measurements. The work function (WF) of PMA for each solvent was calculated using the formula below:

WF = He-I (21.22 eV) − Cut-off energy level of PMA

Cut-off energy levels PMA using acetonitrile, n-Butanol, cyclohexanone, and DMSO were measured at 16.16, 16.20, 16.24, and 16.28 eV, respectively. The calculated WFs of PMA using acetonitrile, n-Butanol, cyclohexanone, and DMSO are 5.06, 5.02, 4.98, and 4.94 eV. The work function of the PMA film was known to be strongly correlated with the reduction of Mo(VI) along with the generation of oxygen vacancies [31]; therefore, the PMA using acetonitrile, which exhibited substantial Mo reduction, showed the smallest work function. As the work function of HIL strongly affects the hole injection characteristics of QD-EL devices, we could expect the device characteristics to also depend on the solvent of PMA.

**Figure 2:**
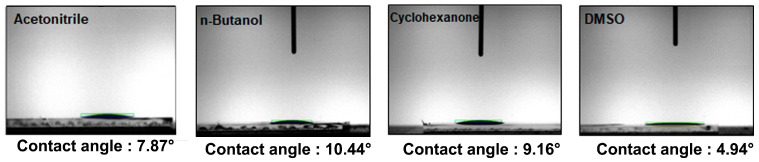
Contact angle of various solvents on ITO substrate.

Figure 2 shows the contact angles of various solvents on the ITO substrate. The contact angle is an important factor in spin coating process. A small contact angle indicates better adhesiveness and wettability. If the contact angle is too large, the coating solution is spread without making a wet film. Therefore, a small contact angle is preferable for thin-film deposition. The contact angles of acetonitrile, n-butanol, cyclohexanone, and DMSO were measured to be 7.87°, 10.44°, 9.16°, and 4.94°, respectively. In terms of contact angle, DMSO appeared to be the most advantageous solvent. However, we need to consider other factors because contact angle does not solely factor into forming a high-quality thin film.

**Figure 3:**
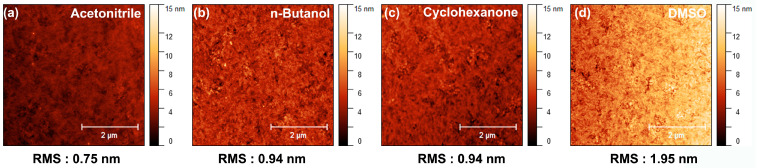
AFM images of PMA thin films with various solvents.

We performed atomic force microscopy (AFM) to confirm the surface morphology of the PMA thin films according to the solvent. (Figure 3) The root-mean-square roughness values of PMA dissolved in acetonitrile, n-butanol, cyclohexanone, and DMSO were 0.75, 0.94, 0.94, 1.95, and 1.95 nm, respectively. Unlike the previous contact angle results, the thin film using DMSO exhibited the worst surface morphology, even though it had excellent adhesiveness and wettability. This result is attributable to the high boiling point of DMSO, which causes slow thin-film formation, resulting in high aggregated morphology. Meanwhile, acetonitrile, n-butanol, and cyclohexanone had moderate RMS roughness values for application to the QD-EL device. Table 1 summarizes the properties of the solvents used.

### 3.2. Device Performance Using PMA as HIL

#### 3.2.1. Electrical Characteristics of Hole-Only Device Using PMA as HIL

**Figure 4:**
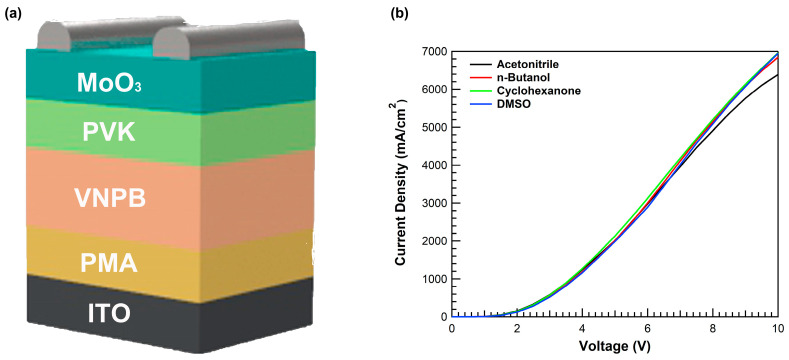
(**a**) Device structure of HOD and (**b**) current density–voltage characteristics using PMA as HIL with various solvents.

Hole-only devices (HODs) were fabricated to evaluate the dependence of the electrical characteristics of PMA as HIL on the process solvent. The HOD was fabricated with the structure of ITO/PMA (<10 nm)/VNPB (40 nm)/PVK (5 nm)/MoO_3_ (10 nm)/Ag (80 nm), as shown in Figure 4a.

Figure 4b shows the current density versus voltage (J–V) curve of the HOD for PMA using various solvents. All devices had similar hole currents for a voltage range of ≤~2.0 V; however, the device using cyclohexanone had a slightly higher current density compared with other devices at a voltage above 2 V. The device using acetonitrile showed the lowest current density at a higher voltage range, which could be due to the high oxygen vacancy of PMA with acetonitrile causing deterioration of the carrier transport properties.

#### 3.2.2. Characteristics of QD-EL Device Using PMA as HIL

**Figure 5:**
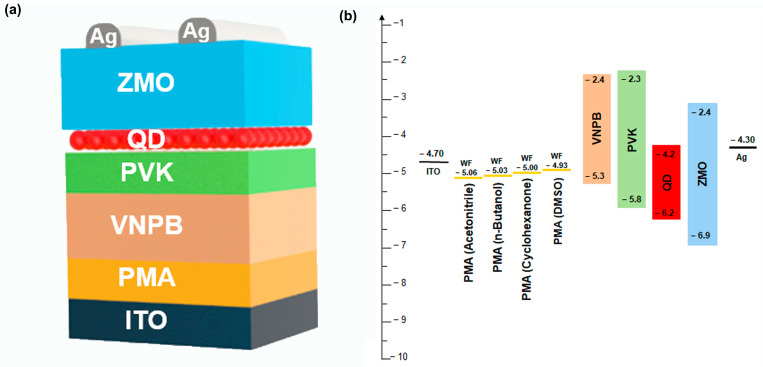
(**a**) Structure of QD-EL device and (**b**) energy band diagram using PMA as HIL with various solvents.

We investigated the effects of the PMA solvent on the performances of QD-EL devices with the structure as shown in Figure 5a. The QD-EL device structure comprised ITO/PMA HIL (< 10 nm)/VNPB (HTL1, 40 nm)/PVK (HTL2, 5 nm)/QDs (emission layer (EML), 20 nm)/Mg-doped ZnO (electron injection/transfer layer (EIL/ETL), 30 nm)/Ag (cathode, 80 nm). We selected the VNPB to HTL1 due to the crosslinking capability by heat treatment. We conducted the solvent washing test of VNPB films with the CB and confirmed its solvent resistance. Figure 5b displays the energy band diagram, lowest unoccupied molecular orbital, and highest occupied molecular orbital energy levels for each layer, and PMA denotes the work function. In general, QD-EL devices exhibit nonradiative Auger recombination due to electron–hole imbalance. Therefore, electron injection should be slowed and hole injection should be improved for the fabrication of high-performance QD-EL devices. For this purpose, Mg-doped ZnO was used instead of conventional ZnO to reduce conductivity and oxygen vacancy, which could cause exciton quenching. Furthermore, for effective hole injection, a double HTL of VNPB and PVK was used for reducing hole injection barriers to balance electrons and holes.

**Figure 6:**
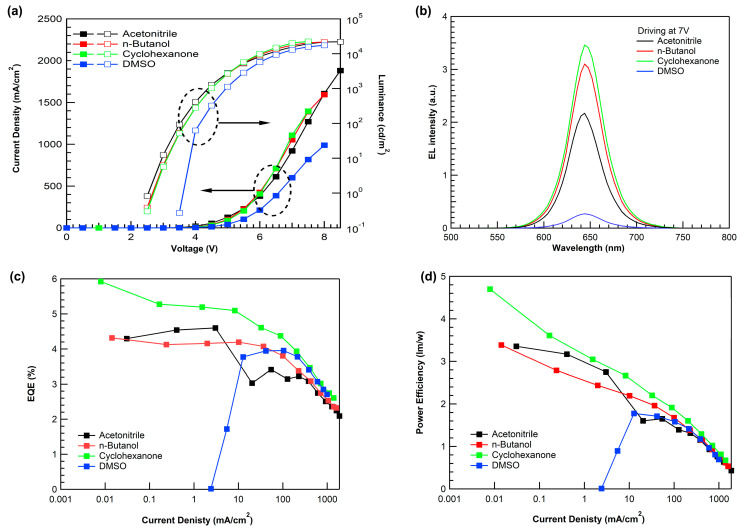
QD-EL device characteristics using PMA with various solvents: (**a**) Current density–voltage–luminance, (**b**) electroluminescence spectra, (**c**) external quantum efficiency–luminance, and (**d**) power efficiency–current density.

Figure 6a shows the current density–voltage–luminance of QD-EL devices using PMA with various solvents. We defined the turn-on voltages (V_on_) as the voltage at the luminance of 1 nit. The V_on_ showed a strong correlation with the work function of the PMA. The PMA with acetonitrile, which exhibited the smallest Von, had an appropriate work function to form an effective cascade structure for hole injection. Therefore, the device using acetonitrile exhibited high current density in the low-voltage region. However, as indicated in previous HOD studies, PMA using acetonitrile had poor current characteristics in the high-voltage region. As a result, the highest EQE_max_ was achieved in the cyclohexanone-based device, which had moderate hole injection and high hole conductivity.

Figure 6b shows a representative electroluminescence spectrum of QD-EL devices at a driving voltage of 7 V. It confirms pure emission from the QD without any parasitic emission, indicating that electrons and holes are sufficiently confined within the QD layer. The maximum external quantum efficiencies (EQE_max_) for the acetonitrile-, n-butanol-, cyclohexanone-, and DMSO-based devices were 4.59%, 4.19%, 5.92%, and 3.95%, respectively. The maximum power efficiency of the QD-EL device also showed a trend similar to EQE_max_, with values of 3.35, 3.38, 4.69, and 1.77 lm/W, respectively. Because of the small work function of the PMA using DMSO, the V_on_ of the device using DMSO had the highest value. Therefore, in terms of the current density, the turn-on current density was higher than the other devices. Table 2 summarizes the QD-EL device performance using various solvents of PMA.

**Figure 7:**
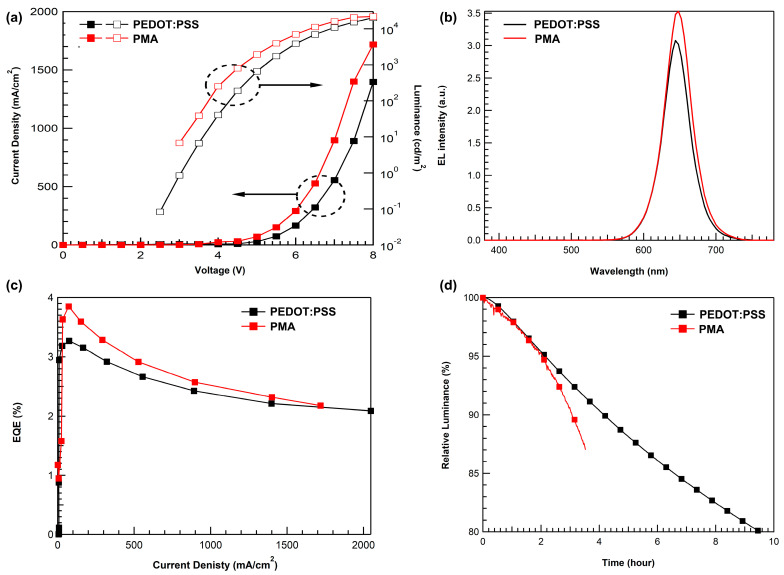
QD-EL device characteristics with PEDOT:PSS and PMA using cyclohexanone: (**a**) current density–voltage–luminance, (**b**) electronic luminance spectra, (**c**) external quantum efficiency–luminance, and (**d**) lifetime test at initial luminance of 1000 nit.

To compare the performance of PMA with the conventional HIL, PEDOT:PSS, we fabricated a device with the same structure except for HIL. As shown in Figure 7a, the PMA-based device had a much higher current density than the PEDOT:PSS-based device. Figure 7a reveals that PMA has better hole injection than PEDOT:PSS, which also improved luminance and resulted in better electrical properties. The electroluminescence spectra in Figure 7b show the same pure QD emission wavelength as shown in Figure 6b. The electroluminescence intensity is also stronger than that of the PEDOT:PSS-based device at the same voltage, as the luminance was enhanced because of the improved electrical characteristics of PMA. The maximum quantum efficiency and power efficiency of the PMA-based device were higher than those of the PEDOT:PSS-based device. Table 3 summarizes the QD-EL device performance comparing PEDOT:PSS and PMA using cyclohexanone. The slightly low performance of the device with PMA using cyclohexanone in Figure 7 compared to that in Figure 6 is due to the experimental deviation. Figure 7d shows the lifetime of the QD-EL device with PEODT:PSS and PMA. The QD-EL device with the PMA had a slightly shorter lifetime than the PEDOT: PSS device. This can be considered as the acidic properties of PMA still deteriorate the device’s stability.

## 4. Conclusions

In summary, we investigated PMA with various solvents for realizing a solution-processed HIL for a QD-EL device. Under inert processing conditions, the solvent affects the reduction behavior of Mo as well as surface morphology. Therefore, oxygen vacancy and work function of the PMA film vary depending on the solvent. Consequently, the behavior of the QD-EL device, such as driving voltage and efficiency, also varies with the process solvent. Moreover, we achieved optimal device performance with cyclohexanone, which have which had moderate work function for hole injection and high hole conductivity.

The optimized PMA HIL provided enhanced device efficiency than PEDOT:PSS, which is a representative solution HIL but still needs further investigation for high stability. However, PMA can provide solvent orthogonality, a simple thin film formation without a high-temperature sintering process or extra process for synthesizing nanoparticles. We believe that polyhetero acid materials could be considered a new material pool for solution-based organic electronic and QD-EL devices.

## Figures and Tables

**Table 1:** Boiling point, viscosity, and surface tension of the solvents used.

Solvent	Boiling Point (°C)	Viscosity (cP)	Surface Tension (dynes/cm)
Acetonitrile	82.1	0.33 (25 °C)	29.29
n-Butanol	117.7	2.54 (25 °C)	24.57
Cyclohexanone	155.6	2.02 (25 °C)	34
DMSO	189	2.0 (25 °C)	43.53

**Table 2:** QD-EL device performances using PMA with various solvents.

Solvent	V_on_ at 1 nit (V)	L_max_ (cd/m^2^)	EQE_max_ (%)	PE_max_ (lm/W)
Acetonitrile	2.5	22,050	4.59	3.35
n-Butanol	2.6	21,700	4.19	3.38
Cyclohexanone	2.7	22,540	5.92	4.69
DMSO	3.6	17,470	3.95	1.77

**Table 3:** Comparing QD-EL device performance with PEDOT:PSS and PMA using cyclohexanone.

HIL	V_on_ at 1 nit (V)	L (cd/m^2^) at 8 V	EQE_max_ (%)	PE_max_ (lm/W)
PEDOT:PSS	2.9	20,390	3.26	1.41
PMA (cyclohexanone)	2.7	22,000	3.80	1.74

## Data Availability

The data presented in this study are available on request from the corresponding author.
